# Congenital solitary kidney in autosomal dominant polycystic kidney disease: Where do known genes end and the unknown begin?

**DOI:** 10.1002/ccr3.7917

**Published:** 2023-11-21

**Authors:** Romina Bucci, Francesca Tunesi, Liliana Italia De Rosa, Paola Carrera, Giulia Mancassola, Martina Catania, Giuseppe Vezzoli, Maria Teresa Sciarrone Alibrandi

**Affiliations:** ^1^ U.O. Nephrology and Dialysis, IRCCS San Raffaele Hospital, Vita Salute San Raffaele University Milan Italy; ^2^ Unit of Genomics for Human Disease Diagnosis, Division of Genetics and Cell Biology IRCCS Hospital San Raffaele Milan Italy

**Keywords:** ADPKD, ciliopathies, genetic, PKD1, URA

## Abstract

We present the case of a 41‐year‐old man patient diagnosed with solitary left kidney with few cysts. He has a family history of unilateral renal agenesis (URA) but no for autosomal dominant polycystic kidney disease (ADPKD). Genetic testing revealed PKD1 gene intron 11 heterozygous nucleotide variant c.2854‐23G>T, but no gene mutation implicated in URA. Just eight cases of ADPKD with one kidney have been recorded globally. PC1 and PC2 disruption, causing primary cilia malformation or absence resulting in relevant in the first embryonic development alteration. Cillia's crucial significance in many diseases will require more research.

## Graphical abstract

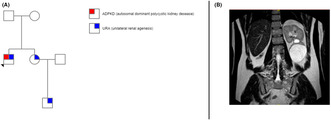

## 1 INTRODUCTION

Autosomal dominant polycystic kidney disease (ADPKD) is the most common monogenic life‐threatening disorder. ADPKD is usually related to changes in the PKD1 and PKD2 genes coding for polycistyn 1 (PC1) and polycistyn 2 (PC2). The disruption of ciliary proteins, including PC1 and PC2, leads to the malformation or absence of primary cilia as a driving force behind the onset of ADPKD. The most common extra‐renal signs of ADPKD are liver and pancreatic cysts, hypertension and vascular alterations.^1^

In ADPKD, kidney and urinary tract anomalies (CAKUT) rarely coexist. Unilateral renal agenesis (URA) is one of the most relevant conditions in the spectrum of the CAKUT.

URA is defined as the one‐sided congenital absence of renal tissue resulting from failure of embryonic kidney formation. The general incidence of URA is 1 in ∼2000.

URA should be distinguished from abnormal or incomplete renal development leading to a non‐functioning kidney, as can be identified in a multicyclic dysplastic kidney (MCDK) or renal aplasia.^2^ MCDK derives from a spontaneous involution of one single kidney.

The occurrence of a solitary kidney in ADPKD has been very rarely described. Only 8 cases of unilateral polycystic kidney disease have been reported in the world.^3^

## 2 CASE REPORT

**FIGURE 1:**
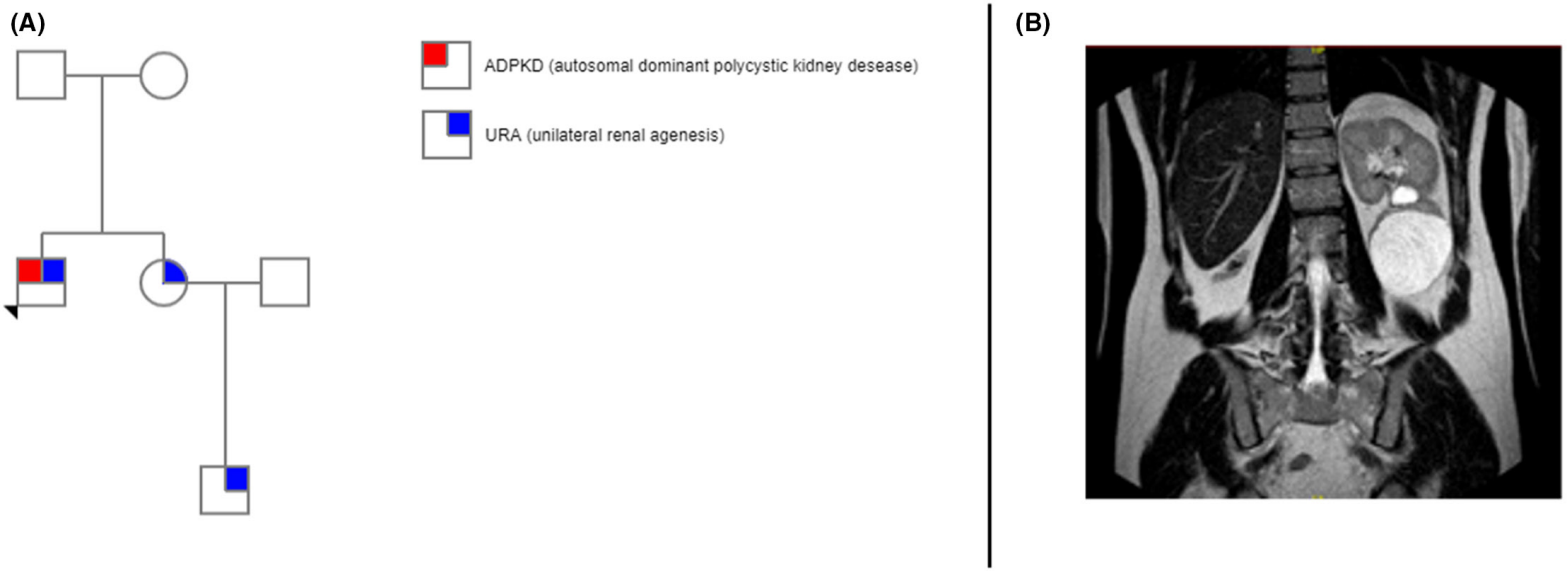
(A) ZG pedigree; (B) magnetic resonance imaging scan of ZG whit solitary left polycystic disease.

A nonsmoking 41‐year‐old male patient (ZG) was diagnosed at the age of 22 years with solitary left kidney with few cysts (Figure 1B), some of them remarkably sized, and no family history of ADPKD.

He had family history of URA as his sister and her son are both suffering from URA but without any cyst (Figure 1A). No other extrarenal ADPKD manifestations, except hypertension treated with ace inhibitor. Renal function was preserved (serum creatinine 0.9 mg/dL, CDK‐EPI 2021 GFR 105 mL/min/1.73 m^2^).

Genetic testing for ADPKD showed the heterozygous nucleotide variation c.2854‐23G>T in intron 11 of PKD1 gene, unreported on official databases. Also, an exome search analysis for URA was performed, but it did not turn up any mutations that might be associated with this condition.

## 3 DISCUSSION

The literature reports only eight cases of ADPKD with a single kidney (Table 1). Poster et al.^3^ described three cases in a cohort of 182 ADPKD patients. All three cases do not have a positive family history for URA, on the contrary ZG has a positive family history for URA but not for ADPKD.

**TABLE 1:** Cases of Unilateral ADPKD reported in the literature.

Sex	Absent kidney	Mutation	Renal function	Reference
Male	Left	NA	ESKD at 58 years	Bear et al.^4^
Female	Right	NA	ESKD at 45 years	Todorov et al.^5^
Male	Left	NA	ESKD at 66 years	Jeong et al.^6^
Male	Left	NA	ESKD at 34 years	Sirvent et al.^7^
Woman	Left	PKD1 c.6487C>T in exon 15	Normal SCr at 24 years	Poster et al.^3^
Men	Left	PDK1 p.W887GfsX11 in exon 11	Normal SCr at 38 years	Poster et al.^3^
Men	Left	NA	Normal SCr at 18 years	Krzemień et al.^8^
Women	Right (hypoplastic)	PDK1 c.12677_ 12678insC in exon 46	Normal SCr at 41 years	Poster et al.^3^

Abbreviations: ADPKD, autosomal dominant polycystic kidney disease; ESKD, end‐stage kidney disease; NA, not available; SCr, serum creatinine.

ZG really caught our interest because of the atypical morphological feature, but the strong history of URA. This failure of symmetry was shared among other members of the pedigree with an inheritance through two generations. No known pathognomonic mutations for URA were found, but PKD1 missense mutation was observed. Curiously, the mother, who does not have the phenotype, also carries the identical mutation.

It's assumed that as a contributing factor to the development of ADPKD, ciliary proteins, such as PC1 and PC2, are disrupted, resulting in the malformation or absence of primary cilia. Cilia proved to be relevant in the first embryonic development stages of many living organisms and of the setting the left–right axis of the body in vertebrates. First of all, cilia serve as antennas for extracellular signaling molecules. Shh19, the vertebrate organogenesis hedgehog protein, is one of these signaling pathways. The other and, in our case, more relevant role, is to set a left–right axis for the body. Interestingly, a total K.O. of PKD2 generates a randomized axis and PKD1L1 (a transient receptor potential channel interacting), mutations can actually lead to left–right asymmetry defects.^9^

More evidence supported primary cilia's crucial roles in several illnesses. Surely other ciliary proteins involved in mechanosensory or chemosensory function must be identified. We can only appreciate the primary cilium's complexity in its many pathogenetic roles if we thoroughly identify every single part within the sensory cilia.

That's why this patient ended up being so interesting: a defect in asymmetry with a concurrent cystic disease, both of them localized only in the kidney. Probably we should look for a different set of genes, not only those strictly related to ADPKD or URA, but also those responsible for the left–right axis, as phenotypic characteristics from various diagnoses may overlap.

ADPKD or ADPKD‐like phenotypes are caused by mutations in other genes much more frequently than commonly assumed. Given the quantity and complexity of genes to be taken into account, a tailored massively parallel sequencing (MPS) approach using a unique, well‐balanced multi‐gene panel should be the most practical and economical approach.

## AUTHOR CONTRIBUTIONS

**Romina Bucci:** Conceptualization; data curation; investigation; writing – original draft; writing – review and editing. **Francesca Tunesi:** Conceptualization; data curation; investigation; writing – original draft; writing – review and editing. **Liliana Italia De Rosa:** Conceptualization; data curation; investigation; writing – original draft; writing – review and editing. **Paola Carrera:** Conceptualization; data curation. **Giulia Mancassola:** Conceptualization; data curation. **Martina Catania:** Conceptualization; data curation; investigation; writing – original draft; writing – review and editing. **Giuseppe Vezzoli:** Conceptualization; writing – original draft; writing – review and editing. **Maria Teresa Sciarrone Alibrandi:** Conceptualization; data curation; supervision; validation; visualization; writing – original draft; writing – review and editing.

## CONFLICT OF INTEREST STATEMENT

All authors declare that they have no conflicts of interest.

## CONSENT STATEMENT

The patient gave written consent to publish this case report and associated images.

## Data Availability

Not applicable. All case#x2010;related data are available as part of the article and no additional source data are required.

## References

[1] Bergmann C , Guay‐Woodford LM , Harris PC , Horie S , Peters DJM , Torres VE . Polycystic kidney disease. Nat Rev Dis Primers. 2018;4(1):50. doi:10.1038/s41572-018-0047-y 30523303 PMC6592047

[2] Schreuder MF , Westland R , Van Wijk JAE . Unilateral multicystic dysplastic kidney: a meta‐analysis of observational studies on the incidence, associated urinary tract malformations and the contralateral kidney. Nephrol Dial Transplant. 2009;24(6):1810‐1818. doi:10.1093/ndt/gfn777 19171687

[3] Poster D , Kistler AD , Krauer F , et al. Kidney function and volume progression in unilateral autosomal dominant polycystic kidney disease with contralateral renal agenesis or hypoplasia: a case series. Am J Kidney Dis. 2009;54(3):450‐458. doi:10.1053/j.ajkd.2009.03.020 19515475

[4] Bear RA . Solitary kidney affected with polycystic disease: a report of 2 cases. J Urol. 1974;111(5):566‐567. doi:10.1016/S0022-5347(17)60016-8 4823963

[5] Todorov VV . The diagnostic dilemma of the unilateral cystic kidney‐ADPKD with aplasia of one kidney. Nephrol Dial Transplant. 1999;14(11):2775. doi:10.1093/ndt/14.11.2775 10534805

[6] Jeong GH , Park BS , Jeong TK , et al. Unilateral autosomal dominant polycystic kidney disease with contralateral renal agenesis: a case report. J Korean Med Sci. 2003;18(2):284‐286. doi:10.3346/jkms.2003.18.2.284 12692431 PMC3055028

[7] Sirvent AE , Enríquez R , Ardoy F , Amorós F , González C , Reyes A . Autosomal dominant polycystic kidney disease with congenital absence of contralateral kidney. Int Urol Nephrol. 2006;38(3–4):773‐774. doi:10.1007/s11255-006-0032-3 17171426

[8] Krzemień G , Turczyn A , Pańczyk‐Tomaszewska M , Jakimów‐Kostrzewa A , Szmigielska A . Long‐term follow up of a boy with unilateral autosomal dominant polycystic kidney disease and contralateral renal agenesis. Dev Period Med. 2017;21(4):380‐383. 29291365 10.34763/devperiodmed.20172104.380383 PMC8522938

[9] Kamura K , Kobayashi D , Uehara Y , et al. Pkd1l1 complexes with Pkd2 on motile cilia and functions to establish the left‐right axis. Development. 2011;138(6):1121‐1129. doi:10.1242/dev.058271 21307098

